# Factors associated with induced abortion among women of reproductive age attending selected health facilities in Addis Ababa, Ethiopia: a case control study

**DOI:** 10.1186/s12905-020-01023-4

**Published:** 2020-09-03

**Authors:** Bikila Soboka Megersa, Oladosu Akanbi Ojengbede, Andreas Deckert, Olufunmilayo Ibitola Fawole

**Affiliations:** 1 Pan Africa University Institute of Life and Earth Sciences (Including Health and Agriculture), University of Ibadan, Ibadan, Nigeria; 2 Arba Minch University, Arba Minch, Ethiopia; 3 Department of Obstetrics and Gynecology, Faculty of Clinical Sciences, College of Medicine, University of Ibadan, Ibadan, Nigeria; 4 Institute of Global Health Heidelberg, Heidelberg University, Heidelberg, Germany; 5 Department of Epidemiology and Medical Statistics, Faculty of Public Health, College of Medicine, University of Ibadan, Ibadan, Nigeria

**Keywords:** Induced abortion, Women of reproductive age, Addis Ababa, Ethiopia

## Abstract

**Background:**

There has been a significant reduction of abortion rates in high-income countries, while the rates remain unchanged in low- and middle-income countries. In Ethiopia, for example, the number of women of reproductive age seeking an induced abortion is increasing. However, there is limited information concerning the reasons why the occurrence of this procedure is increasing. Thus, this study aimed to identify factors associated with having induced abortion in Addis Ababa, Ethiopia.

**Methods:**

An unmatched case-control study was conducted using a semi-structured, interviewer-administered questionnaire from October to December 2017. The cases were 147 women of reproductive age who underwent abortion in a health facility or presented with complications due to induced abortion conducted outside the health facility. The controls were 295 women who came for antenatal care and who reported never having had an induced abortion. The cases were selected by consecutive sampling from nine health facilities, whereas the controls were selected by systematic sampling from the same health facilities. Bivariate and multivariate logistic regression models were employed using STATA version 14 to identify factors associated with induced abortion.

**Results:**

The mean age of cases was 26.5 ± 5.7 years, while for the controls it was 28.1 ± 4.8 years. Being unmarried (AOR = 9.6; 95% CI: 1.5–61.7), having primary (AOR = 5.3; 95% CI: 1.5–18.3) and tertiary (AOR = 5.7; 95% CI: 1.6–21.1) education, earning monthly income 100–300 USD (AOR = 0.2; 95% CI: 0.1–0.4) and >  300 USD (AOR = 0.1; 95% CI: 0.0–0.2), initiating first intercourse between ages of 15 and 19 (AOR = 4.7; 95% CI: 1.4–15.6), marrying before the age of 18 (AOR = 2.9; 95% CI: 1.3–6.7), and having two children (AOR = 4.7; 95% CI: 1.8–12.7) were independent predictors of induced abortion.

**Conclusion:**

Family planning programs hoping to reduce the occurrence of induced abortion should specifically target unmarried women, low income, and those who have two children. The government should also work on preventing early marriage and providing sexual and reproductive health education to help adolescents delay age at first sexual experience.

## Background

Induced abortion is described as surgical or medical termination of a live fetus before the time of fetus viability [1]. According to the World Health Organization (WHO), induced abortion can be safe or unsafe [2]. Unsafe abortion causes a significant proportion of maternal deaths and morbidity each year, 22 million unsafe abortions are estimated to take place globally and it is a leading cause of morbidity and mortality in many sub-Saharan African countries [3, 4].

Globally, from 2010 to 2014 25% of pregnancies ended in abortion and the global annual rates of abortion were estimated at 35 abortions per 1000 women of childbearing age. There has been a significant reduction of abortion rates in high-income countries, while the rates remain unchanged in low- and middle-income countries [5]. The WHO reported that Africa accounts for 1.7 million of the estimated 5 million women of reproductive age hospitalized annually worldwide for treatment of abortion-related complications. Deaths from induced unsafe abortion are known to contribute to approximately 14% of all maternal deaths in Africa [3].

The danger from unsafe abortion was recognized by the Ethiopian Parliament in 2005 when evidence indicated it contributed 32% of the burden of maternal mortality in Ethiopia. This prompted amendments to the penal code on abortion which allows safe abortion to be performed legally in cases of rape or incest, if the woman has physical or mental disabilities, if it is needed to preserve the woman’s life or her physical health, or if she is a minor who is physically or mentally unprepared for childbirth. Prior to the 2005 reforms, abortion was only allowed to save the life of the pregnant woman. Since the enactment of the new law, the Ethiopian Ministry of Health has led the expansion of comprehensive abortion care in governmental and private health facilities [6].

The legal abortion service provided in both governmental and private health facilities in Ethiopia is guided by the Technical and Procedural Guideline for Safe Abortion Service. This guideline has explained the termination of pregnancy by either medical or surgical abortion dependent on the gestational age since the last menstrual period (LMP), level of health care, and professional knowledge. At the primary health care level, there are two methods which allow for the provision of safe abortion depending on the gestational age. Medical termination of the pregnancy is used if the gestational age is less than 9 completed weeks since the LMP and vacuum aspiration is used up to 12 weeks from the LMP. The guideline explained the treatment of complicated cases of post-abortion in both general and referral hospitals [7].

Based on a nationwide estimate of induced abortion in Ethiopia, most of the safe abortion services were provided by non-governmental health facilities, while the majority of post-abortion care was delivered by government hospitals and health centers in 2014. The annual average caseload of safe abortion cases was estimated 151 cases per facility. In Ethiopia, the safe induced abortion rate increased from 22 to 28 per 1000 women of reproductive age between 2008 and 2014. On the other hand, the number of women seeking treatment for post-abortion complications increased from 52,600 in 2008 to 103,600 cases in 2014. Although the proportion of abortions occurring in health facilities increased from 27% in 2008 to 53% in 2014, an estimated 294,127 abortions (47% of all abortions) still occurred outside of health facilities [8]*.*

After the amendment of Ethiopia’s abortion law in 2005, post-abortion complications from induced abortion continued as the significant health burden of women in the reproductive age group. A nationwide study on abortion related morbidity has shown that 58,000 women in reproductive age visited health facilities seeking post-abortion care in 2008 and more than 13,000 were admitted for the treatment of abortion complications [9]. Research has shown that the high burden of post-abortion complications consumed a large portion of the reproductive health budget in Ethiopia [10].

Addis Ababa has the highest contraceptive utilization rate and the lowest unmet need for family planning among the regions of Ethiopia [11]; still, the highest prevalence of induced abortion occurred in Addis Ababa [12]. Though abortion laws have been eased, they are still restrictive and in view of the morbidity and mortality associated with complications of unsafe induced abortion, it is important to identify abortion seekers so that it can be prevented. Therefore, this study aimed to identify factors associated with having induced abortion in Addis Ababa, Ethiopia.

## Methods

### Study area

This study was conducted in Addis Ababa, the largest city and the capital of Ethiopia. According to the Central Statistics Agency of Ethiopia, the total inhabitants of Addis Ababa was estimated to be 4 million in 2016 [13]. Administratively the city is divided into 10 sub-cities, 116 districts, and 203 kebeles (administrative unit). The city has 14 public hospitals, 37 private hospitals, 84 public health centers, and around 700 private clinics [14].

### Study design

The study design was an unmatched case-control study. The source populations for this study were women of reproductive age who were (at the age of 16–49 years) living in Addis Ababa, Ethiopia.

### Study population

The cases were women of reproductive age who underwent abortion in a health facility or presented with complications due to induced abortion conducted outside the health facility. The controls were women of reproductive age who presented to the health facility for antenatal care and who reported never having had induced abortion previously. We included women of reproductive age who could autonomously sign the informed consent, therefore, were in the age range between 16 and 49.

### Sample size

The sample size was determined using a formula for two population proportions and was calculated using Open Epi version 2.3 statistical software. The contraceptive utilization among currently married women in Addis Ababa is 56% [11]*.* This figure, as a percent of controls exposed to contraception (main exposure variable), was considered to detect an odds ratio (OR) of 0.55 [15] with a 95% confidence interval, 80% power of the study, and a case control ratio of 2:1. A 10% adjustment was made for non-response. Thus, a minimum sample size of 134 cases and 268 controls was calculated; finally, 147 and 295 questionnaires were collected for cases and controls respectively.

### Sampling technique

#### Cases

From the 10 sub-cities of Addis Ababa three sub-cities: Arada, Lideta, and Kirkos were selected by simple random sampling by balloting. The health facilities in each sub-city were stratified into two categories, governmental and non-governmental. From the list of all health facilities in each sub-city, two governmental (one hospital and one health center) and one non-governmental health facility which provides abortion services were selected randomly by balloting. The number of respondents interviewed was proportionally allocated between nine health facilities (six governmental and three non-governmental) using the average number of cases of induced abortion managed in the past 3 months as a guide. Respondents were selected by consecutive sampling.

#### Controls

The controls were selected from the same (three) health facilities used for selecting the cases. The number of respondents to be interviewed was proportionally allocated to the selected health facilities based on average attendance of antenatal care in the last 3 months. Systematic random sampling was used to select respondents at each of the health facilities. To identify the first respondent the lottery method was employed.

### Data collection tools and methods

Data were collected using an interviewer-administered, semi-structured questionnaire from October to December 2017. The questionnaire had 20 items divided into three sections. It obtained information on respondent’s socio-demographic characteristics, reproductive health behaviours, and contraceptive use. The questionnaire was developed after extensive review of relevant studies which assess determinants of induced abortion in low- and middle-income countries, adjusted to the local context of the study [15–20]. The questionnaire was prepared in English and then translated into the local language (Amharic) for the data collection. The questionnaire was pretested in Adama town on 10% of the sample size (15) for cases and (30) for controls. The results of the pretest were not considered in the final analysis, but corrections were made accordingly to some of the questions to make it easy to understand.

Ten nurses working in the antenatal clinic and eight midwives from the abortion service were trained on data collection. The interviewers were trained on how to administer the questionnaire and on maintaining the confidentiality of respondents’ information by the investigators over a period of 3 days. The interview took about 30 min and it was undertaken in a consulting room where privacy was secured. The data collection was supervised by three public health professionals, who checked the collected data daily for completeness and accuracy. Incomplete or unclear questionnaires were immediately returned to the interviewers for completion or correction. To minimize misclassification, a history of previous abortion was asked from the women who came for antenatal care and checked with their medical records to ensure they are true controls.

### Study variables

The outcome variable was the abortion status of women. The independent variables were their socio-demographic characteristics, reproductive health behaviours, and contraceptive use. The rationale for variable inclusion was based on review of relevant literature on induced abortion [15, 16, 19–21].

### Data analysis

The data were checked and entered using Epi-data version 4.0.2.49 and imported to Stata version 13 for analysis. Descriptive statistics using the measure of central tendency and proportions were used to describe the characteristics of the study population. Chi-square tests were used to measure the association between the predictor variables and induced abortion. In unadjusted model, bivariate logistic regression was used to determine the crude odds ratio (COR) of induced abortion with each independent variable. The significant variables in bivariate logistic regression were entered into a multivariable logistic regression model to adjust for the other potential confounders. Multivariable logistic regression analysis was used to present the adjusted odds ratio (AOR) of having an induced abortion with the independent variables. The odds ratio was determined at 95% confidence interval (CI) in the logistic regression analysis. A *p*-value less than 0.05 was considered statistically significant.

## Results

### Socio-demographic characteristics of the respondents

A total of 442 participants, 147 (33.3%) of cases and 295 (66.7%) controls completed the interview, resulting in a response rate of 98.9%. The main reason given for not completing the interview was related to the sensitive nature of the questions. The mean age of the respondents was 27.6 ± 4.1 years (26.6 ± 5.7 for cases and 28.1 ± 4.8 for controls). Ninety-two (62.6%) of cases and 279 (84.0%) controls were married. The predominant religion was Orthodox Christian for both cases and controls. About half of the cases and the controls had tertiary education. The majority of the cases and controls were employed, while 21.1% of the cases and 7.8% of controls were students. Ninety-three (63.3%) of the cases and about one-fourth (24.8%) of the controls earned less than 100 dollars (USD) per month (Table 1).

**Table 1:** Socio-demographic characteristics of women in the reproductive age attending selected health facilities, Addis Ababa, Ethiopia, 2017

Variables	Cases		Controls		chi^**2**^ ***p***-value
	Frequency	Percent	Frequency	Percent	
**Age group (years)**					
16–19	14	8.8	15	5.4	0.017
20–24	39	26.5	53	18.0	
25–29	58	39.5	114	38.6	
30–34	21	14.3	79	26.8	
≥35	16	10.9	33	11.2	
**Marital status**					
Married	92	62.6	279	94.6	< 0.001
Unmarried	55	37.4	16	5.4	
**Ethnicity**					
Amhara	50	34.0	116	39.3	0.190
Oromo	50	34.0	73	24.8	
Tigre	16	10.9	39	13.2	
Gurage	28	19.1	53	18.0	
Others^b^	3	2.0	14	4.7	
**Religion**					
Orthodox	107	72.8	186	63.1	0.111
Muslim	22	14.9	64	21.6	
Protestant	15	10.2	30	10.2	
Catholic	1	0.7	15	5.1	
Others^c^	2	1.4	0	0.0	
**Educational status**					
No formal education	7	4.8	37	12.5	< 0.001
Primary	36	24.5	33	11.2	
Secondary education	32	21.7	84	28.5	
Tertiary	72	49.0	141	47.8	
**Occupation**					
Employed	60	40.8	132	44.8	< 0.001
Housewife	34	23.1	121	41.0	
Student	31	21.1	23	7.8	
Daily laborer	22	15.0	19	6.4	
**Monthly income**					
< 100 USD	93	63.2	73	24.8	< 0.001
100–300 USD	42	28.6	160	54.2	
>300 USD	12	8.2	62	21.0	

^b^Silte, Wolayita, Hadiya, and Sidama

^c^Adventists, Jehovah Witness, and Wakeffata

### Reproductive health behaviour of the respondents

About half of the cases (51.0%) and 38.3% of controls were between 15 and 19 years of age at first intercourse. Twenty-two (23.9%) of the cases and 21 (7.5%) controls were married before the age of 18. Fifty-nine (40.1%) of cases and 87 (29.5%) of controls had one pregnancy in their lifetime. Sixty-nine (46.9%) of cases and one-third (33.2%) of controls had not had any children. Sixty-one (41.5%) of the cases and 136 (46.1%) of controls had two or more sexual partners in a lifetime. About one-third, 47 cases and 91 controls had two or more sexual partners in the past 12 months (Table 2).

**Table 2:** Reproductive health behaviour of women in the reproductive age attending selected health facilities, Addis Ababa, Ethiopia, 2017

Variables	Cases		Controls		chi^**2**^ ***p***-value
	Frequency	Percent	Frequency	Percent	
**Age at first intercourse**					
15–19	75	51.0	113	38.3	0.013
20–24	63	42.9	144	48.8	
≥ 25	9	6.1	38	12.9	
Total	147	100.0	295	100.0	
**Age at first marriage**					
< 18	22	23.9	21	7.5	0.001
≥ 18	70	76.1	258	92.5	
Total	92	100.0	279	100.0	
**Number of lifetime pregnancies**					
1	59	40.1	87	29.5	0.081
2	42	28.6	100	33.9	
≥3	46	31.3	108	36.6	
Total	147	100.0	295	100.0	
**Number of children**					
0	69	46.9	98	33.2	0.002
1	42	28.6	115	39.0	
2	26	17.7	37	12.5	
≥ 3	10	6.8	45	15.3	
Total	147	100.0	295	100.0	
**Number of sexual partners in lifetime**					
1	86	58.5	159	53.9	0.359
≥ 2	61	41.5	136	46.1	
Total	147	100.0	295	100.0	
**Number of sexual partners in the past 12 months**					
1	100	68.0	204	69.2	0.810
≥ 2	47	32.0	91	30.8	
Total	147	100.0	295	100.0	

### Use of family planning method

About 95% of the cases and controls could name at least one family planning method. About three-fourth of the cases (76.2%) and controls (74.2%) had ever used a family planning method. The pill was used as their most recent family planning for both cases and controls (35.7 and 28.3%, respectively). Of the women who did not use family planning methods, 45.7% of cases reported because of partners’ disapproval, while about half (52.0%) of the controls were not using family planning to become pregnant (Table 3).

**Table 3:** Family planning utilization of women in the reproductive age attending selected health facilities, Addis Ababa, Ethiopia, 2017

Variables	Cases		Controls		chi^**2**^ ***p***-value
	Frequency	Percent	Frequency	Percent	
**Knowledge of any family planning method**					
Yes	142	96.6	282	95.6	0.614
No	5	3.4	13	4.4	
Total	147	100.0	295	100.0	
**Ever use of any family planning method**					
Yes	112	76.2	219	74.2	0.656
No	35	23.8	76	25.8	
Total	147	100.0	295	100.0	
**Family planning method last used**					
Condom	20	17.9	23	10.5	0.112
Pill	40	35.7	62	28.3	
Intra uterine device	10	8.9	38	17.4	
Injectable	15	13.4	44	20.1	
Implants	17	15.2	36	16.4	
Breast feeding	2	1.8	4	1.8	
Calendar method	8	7.1	12	5.5	
Total	112	100.0	219	100.0	
**Reason for not using family planning**					
Want to get pregnant	5	14.3	40	52.0	0.201
Fear family planning may affect health	14	40.0	13	17.3	
My partner does not allow me to use	16	45.7	23	30.7	
Total	35	100.0	76	100.0	

### Factors associated with induced abortion

Bivariate analysis showed respondent’s age, marital status, educational status, occupation, monthly income, age at first intercourse, age at first marriage, and number of children had significant associations with having an induced abortion.

Women between the ages of 30 and 34 were less likely to report having induced abortion than those who were aged 16–19 (COR = 0.3; 95% CI: 0.1–0.7). The odds of having an induced abortion was higher among unmarried women compared with those who were married (COR = 10.4; 95% CI: 5.7–19.1). Women who had primary and tertiary education were more likely to have an induced abortion compared with those who had no formal education (COR = 5.8; 95% CI: 2.3–14.7) and (COR = 2.7; 95% CI: 1.1–6.4) respectively. Women who were students and daily laborers were more likely to report having an induced abortion than employed women (COR = 3.0; 95% CI: 1.6–5.5) and (COR = 2.5; 95% CI: 1.3–5.1) respectively. Women whose monthly income was from 100 to 300 USD and more than 300 USD were less likely to report having an induced abortion compared with those who were less paid (AOR = 0.2; 95% CI: 0.1–0.3) and (AOR = 0.2; 95% CI: 0.1–0.3) respectively.

The odds of induced abortion among women who had first intercourse between the ages of 15 and 19 were 2.8 times higher than those who had first intercourse at age of 25 years or later (AOR = 2.8; 95% CI: 1.3–6.1). Women who married before the age of 18 were 3.0 times more likely to report having an induced abortion compared with those who married at the age of 18 or later (AOR = 3.0; 95% CI: 1.5–5.9). The odds of having an induced abortion was less likely among women who had one child and those who had three or more children compared with those who had no child (COR = 0.5; 95% CI: 0.3–0.8) and (COR = 0.7; 95% CI: 0.5–0.9) respectively (Table 4).

**Table 4:** Bivariate and multivariable logistic regression of predictors of induced abortion among women in the reproductive age attending selected health facilities, Addis Ababa, Ethiopia, 2017

Variables	COR (95% CI)	***P***- value	AOR (95% CI) ^**a**^	***P***-value
**Age group (Years)**				
16–19	1.0		1.0	
20–24	0.9 (0.4–2.1)	0.817	0.6 (0.1–2.8)	0.510
25–29	0.6 (0.3–1.4)	0.250	1.3 (0.3–6.4)	0.750
30–34	0.3 (0.1–0.7)	0.012	1.6 (0.3–8.3)	0.603
≥35	0.6 (0.2–1.5)	0.284	3.9 (0.6–23.1)	0.137
**Marital status**				
Married	1.0		1.0	
Unmarried	10.4 (5.7–19.1)	< 0.001	9.6 (1.5–61.7)	0.017
**Educational status**				
No formal education	1.0		1.0	
Primary	5.8 (2.3–14.7)	< 0.001	5.3 (1.5–18.3)	0.008
Secondary	2.0 (0.8–5.0)	0.129	2.3 (0.7–7.6)	0.181
Tertiary	2.7 (1.1–6.4)	0.023	5.7 (1.6–21.1)	0.008
**Occupation**				
Employed	1.0			
Housewife	0.6 (0.4–1.0)	0.053	0.9 (0.4–2.0)	0.885
Student	3.0 (1.6–5.5)	0.001	2.3 (0.6–8.8)	0.238
Daily laborer	2.5 (1.3–5.1)	0.008	0.8 (0.3–2.5)	0.664
**Monthly income**				
< 100 USD	1.0		1.0	
100–300 USD	0.2 (0.1–0.3)	< 0.001	0.2 (0.1–0.4)	< 0.001
≥ 300 USD	0.2 (0.1–0.3)	< 0.001	0.1 (0.0–0.2)	< 0.001
**Age at first intercourse**				
15–19	2.8 (1.3–6.1)	0.010	4.7 (1.4–15.6)	0.011
20–24	1.8 (0.8–4.1)	0.125	2.1 (0.7–6.6)	0.190
≥ 25	1.0		1.0	
**Age at first marriage**				
< 18	3.0 (1.5–5.9)	0.001	2.9 (1.3–6.7)	0.011
≥ 18	1.0		1.0	
**Number of children**				
0	1.0		1.0	
1	0.5 (0.3–0.8)	0.006	1.9 (0.8–4.5)	0.127
2	1.0 (0.6–1.8)	0.995	4.7 (1.8–12.7)	0.002
≥3	0.7 (0.5–0.9)	0.003	0.9 (0.3–3.0)	0.883

*Note*: *CI* confidence interval, *COR* crude odds ratio, *AOR* adjusted odds ratio

^a^Final model adjusted for all variables shown in the table

In the multivariable analysis, marital status, educational status, monthly income, age at first intercourse, age at first marriage, and number of children remained statistically significant. The odds of having an induced abortion was higher among unmarried women compared with those who were married (AOR = 9.6; 95% CI: 1.5–61.7). Women who had primary and tertiary education were more likely to report having an induced abortion than those who had no formal education (AOR = 5.3; 95% CI: 1.5–18.3) and (AOR = 5.7; 95% CI: 1.6–21.1) respectively. Women whose monthly income was from 100 to 300 USD and more than 300 USD were less likely to report having an induced abortion compared with those who were less paid (AOR = 0.2; 95% CI: 0.1–0.4) and (AOR = 0.1; 95% CI: 0.0–0.2) respectively.

The odds of induced abortion among women who had first intercourse between the ages of 15 and 19 were 4.7 times higher than those who had first intercourse at the age of 25 or older (AOR = 4.7; 95% CI: 1.4–15.6). Women who married before the age of 18 were 2.9 times more likely to report having an induced abortion compared with those who married at 18 years or later (AOR = 2.9; 95% CI: 1.3–6.7). Women who had two children had 4.7 higher odds of induced abortion than those who had no child (AOR = 4.7; 95% CI: 1.8–12.7) (Table 4).

## Discussion

This study is one of the first quantitative studies to compressively identify factors associated with induced abortion among women of reproductive age in Addis Ababa, Ethiopia. In multivariable logistic regression model marital status, educational status, monthly income, age at first intercourse, age at first marriage, and women’s number of children were independently strongly associated with having an induced abortion.

We found that unmarried women were more likely to report having an induced abortion compared with those who were married. This is similar to findings from other studies in Ethiopia [16, 19, 20, 22], reporting that abortion seekers were more likely to be unmarried (single or divorced or widowed). This could be due to a tendency of delay in women’s age at marriage in Addis Ababa [23–25] which have been suggested to result in an increased sexual activity among unmarried women and raising their risk of unintended pregnancy [18, 23, 24, 26]. These women also may obtain an induced abortion because they feel that having a child would interfere with future opportunities [27]. Yet, fear of school dropout was mentioned as a primary reason for demanding an induced abortion service among women of reproductive age in Ethiopia [20, 28]. Similarly, studies conducted in low- and middle-income countries have consistently shown that unmarried women (single/separated/divorced/widowed) had higher odds of having an induced abortion compared with those who were married [15, 29, 30]. Other reasons could be difficulty in accessing contraception, not having adequate financial and social support to provide for their unborn child, and stigma related to having a child outside of wedlock have been indicated for higher odds of induced abortion among unmarried women [27, 31, 32].

Women with primary and tertiary education had higher odds of having an induced abortion compared with women without formal education. This finding is consistent with the results of other studies conducted in Addis Ababa [33], East and Northwest Ethiopia [16, 34]. This could be due to better educated women may have improved access to health information [35] and thus, better knowledge of the abortion law [36]. Similarly, the evidence provided by other studies from Ghana [29, 36, 37], Nigeria [38], Kenya [39], Iran [40] have shown that better educated women had higher odds of having an induced abortion compared with less educated women. The other possible reasons for higher odds of induced abortion among more educated women may be attributed to their employment status and lack of time to care for children [41], wanting to postpone/space childbirth [27], and more tendency to plan their family size [42]. However, our study contradicts the findings of other studies conducted in Ethiopia [19, 28] and elsewhere [21, 43, 44] which have reported the higher the education of the women the lower was the rate of induced abortion. This dissimilarity may be attributed to the differences in socio-demographic characteristics of the respondents and the setting from which the study respondents were selected.

Women with lower income had higher odds of having an induced abortion as compared to those with higher income, which is consistent with the evidence provided by other studies from Ethiopia [20, 33]. Finding from the Ethiopian Demographic and Health Survey has indicated that use of modern contraception increases sharply with wealth, ranging from 20% for women in the lowest wealth quintile to 47% for women in the highest wealth quintile [11]. Thus, the low contraceptive utilization among women with lower income may account for the higher odds of induced abortion in this study. In turn, studies have demonstrated that women who utilize contraceptives are less likely to have an induced abortion as compared to those who did not use [16, 45–47]. The finding of this study is also in line with other studies conducted elsewhere [27, 48, 49]. Conversely, studies have reported higher odds of having an induced abortion among women of high-income status [18, 21, 29, 37, 50, 51]. Another explanation for higher odds of having an induced abortion among lower income women could be that these women may not be financially prepared to raise their unborn child and it could affect their future opportunities [27]. It has also been suggested that women with lower income might experience a higher number of unintended pregnancies compared with those with higher income counterparts [49].

In this study, we found that women who had their first sexual experience between the ages 15 and 19 were more likely to report having an induced abortion compared with those who had their first intercourse at the age of 25 or later. Likewise, other studies from Ethiopia [17] and elsewhere [52–54] have reported the higher likelihood of having an induced abortion among women who had their sexual debut at younger ages. This could be because these women have more years of exposure to the risk of experiencing an unintended pregnancy [55, 56]. Another possible explanation could be that women who initiated their first sexual intercourse at an early age may have limited awareness and financial access to use family planning. Thus, the government should work to help adolescents delay age at the first sexual experience and encourage family planning use hoping that it could reduce induced abortion.

Women who married before the age of 18 had higher odds of having an induced abortion than those who married at the age of 18 or later. This finding is also consistent with the evidence provided by other studies [40, 57]. A possible reason is that women who marry at earlier ages may complete their ideal family size earlier and could have a higher likelihood of unintended pregnancy in their lifetime [34]. In turn, studies have consistently shown that unintended pregnancy increases the likelihood of induced abortion [58, 59]. Thus, the government should focus on the prevention of early marriage among adolescents.

In this study, women with two children were more likely to report having an induced abortion compared with women who had no child. Likewise, studies conducted in Ethiopia have shown that the likelihood of terminating a pregnancy was higher among women having multiple pregnancies [16, 19, 28]. Women who have their ideal family size may choose to terminate an unwanted pregnancy to restrict their family size [28, 40, 42]. The Ethiopian Demographic and Health Survey report has indicated that women of reproductive age in Addis Ababa had the lowest ideal family size (3.6 children) and fertility rate (1.8) in the country [11]. In addition, studies have revealed that the fertility rate in Addis Ababa has been declined below replacement level which is attributed to economic, demographic, and social reasons [23, 25]. Therefore, women in Addis Ababa who desire to have a small family size may obtain an induced abortion in order to space birth intervals or limit births. Likewise, studies have reported a disproportionate share of induced abortions occurred among women who had several pregnancies suggesting that these women obtain abortions to space birth intervals or limit births as well [18].

One of the strengths of this study is the very high response rate. Additionally, our sample size was informed by a sample size calculation and we were able to exceed the limit of the calculated sample size, which did not have any ethical implications since the study was observational only but increased the precision of our estimates. Another strength of our study is that the cases and controls were recruited randomly from the same health facilities; also, the health facilities were selected randomly.

The study, however, has some limitations. There is a concern for information bias as women’s history of induced abortion was self-reported and therefore some controls may have actually had abortions in the past but wanted to give responses they felt were socially desirable. However, respondents were encouraged to tell the truth, and confidentiality was assured. The medical record of controls was then cross-checked to ensure they had not procured an abortion. Secondly, the study was health facility-based, therefore, the results may not be extrapolated to represent the general women population of Addis Ababa.

Thirdly, the analysis did not control for facility-level factors which could introduce some bias; however, the health facilities were selected randomly which most probably decreased potential confounding on a facility level. Fourthly, our cases might not completely represent all induced abortions. Many induced abortions happen outside the health facilities and the cases never get recognized. The characteristics of women who undergo abortion outside the facilities might differ from those who received induced abortion services at the facilities. However, we included women who underwent induced abortion outside the facilities but presented at the facilities for treatment related to the complications of induced abortion. With this approach, we could partially cover induced abortions that occurred outside the facilities.

Fifthly, the controls may not represent the same population from which the cases were recruited. The controls consisted of women who came for antenatal care at the facilities; population controls were not possible due to ethical issues and feasibility. And finally, the results might not be generalizable for women under the age of 16 since they were not included due to legal issues.

## Conclusion

This study has identified characteristics of women who induce abortion which may reduce maternal morbidity, mortality, and the health care burden of post-abortion complications from unsafe abortion in Ethiopia (Addis Ababa). Sexual and reproductive health education, and family planning programs should target unmarried women, women with high educational status, low-income women, those who initiated sexual intercourse and married at a younger age, and those with a desire to limit childbearing considering it could reduce induced abortion.

## Data Availability

The data sets generated and/or analyzed during the current study are not publicly available due to confidentiality. However, someone who wants to have the data can access from the corresponding author.

## Abbreviations

AOR: Adjusted Odds Ratio
COR: Crude Odds Ratio
CI: Confidence Interval
LMP: Last Menstrual Period
USD: United States Dollar
WHO: World Health Organization
